# Crystal-induced acute kidney injury due to ciprofloxacin

**DOI:** 10.12860/jnp.2015.06

**Published:** 2015-01-01

**Authors:** Mahboob Khan, Luis M Ortega, Nasreen Bagwan, Ali Nayer

**Affiliations:** ^1^Division of nephrology and Hypertension, Allegheny General Hospital, Temple University School of Medicine, Pittsburgh, PA, USA; ^2^Department of Medicine, Allegheny General Hospital, Pittsburgh, PA, USA; ^3^Division of Nephrology and Hypertension, University of Miami, Miami, FL, USA

**Keywords:** Acute kidney injury, Interstitial nephritis, Fluoroquinolones, Crystalluria

## Abstract

*Background:* Fluoroquinolones are known to cause acute renal failure due to interstitial nephritis.

*Case Presentation:* Here we present an elderly woman who developed oliguric acute kidney injury (AKI) after receiving oral and intravenous ciprofloxacin in a 48-hour period. Recently, several case reports have been published in the literature regarding the presence of crystals in the urine sediment of patients treated with ciprofloxacin for different types of systemic infections. Ciprofloxacin crystals precipitate in alkaline urine and provoke renal failure through intra-tubular precipitation.

*Conclusions:* Conservative measures including intravenous hydration and avoidance of alkalinization of the urine can reverse this condition if applied in time.

**Implication for health policy/practice/research/medical education:**

Fluoroquinolones are known to cause acute renal failure due to interstitial nephritis. The crystals of this agent, precipitate under alkaline urine and provoke renal failure through intra-tubular precipitation. Conservative measures, that include hydration with standard intravenous fluid formulations and avoidance of alkalinization of the urine, can reverse this condition if applied on time.

## 1. Introduction

Ciprofloxacin is an antimicrobial fluoroquinolone. Acute kidney injury (AKI) as a result of tubulointerstitial nephritis due to this drug has been described (1) Ciprofloxacin causes crystal nephropathy in experimental animals (2). Crystalluria due to ciprofloxacin has been recorded in two out of 63,000 patients (3). Unfortunately very few images of ciprofloxacin crystals are available in the literature (4). The crystals show a wide array of morphological appearances and precipitates in a urine pH> 6.8. The likelihood of occurring in humans with intact tubular function is low (4). Here we present a case of an elderly patient that developed oliguric AKI after receiving oral and intravenous ciprofloxacin in a 48-hour period. Previous cases have been described after receiving oral ciprofloxacin in a 24-hour period or after 8 days of therapy (5,6). To prevent crystalluria, patients should be well hydrated,and urine alkalization should be avoided (7).

## 2. Case Presentation

**Figure 1:**
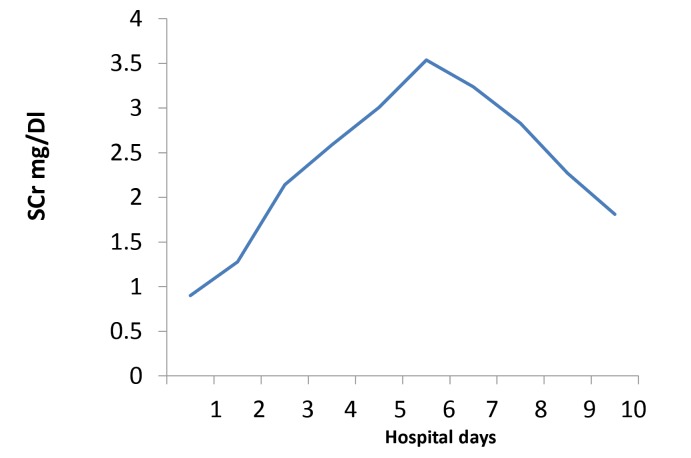
Serum creatinine trend

**Figure 2:**
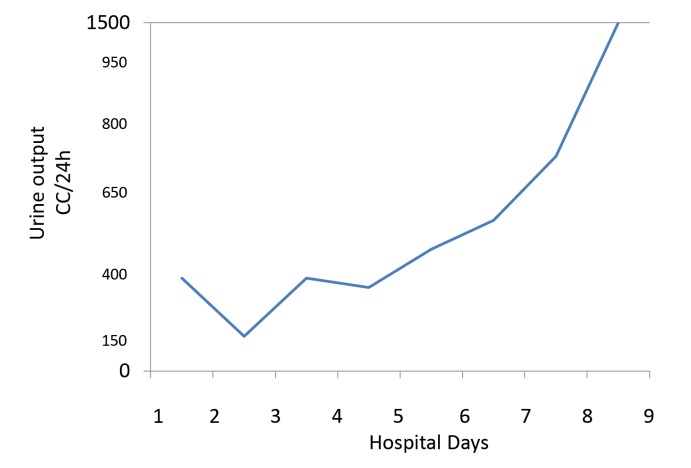
Urine output

**Figure 3:**
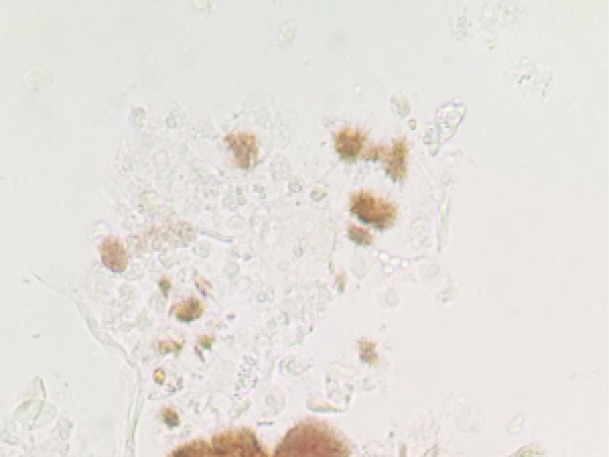
Stellate-shaped ciprofloxacin crystals at low power

**Figure 4:**
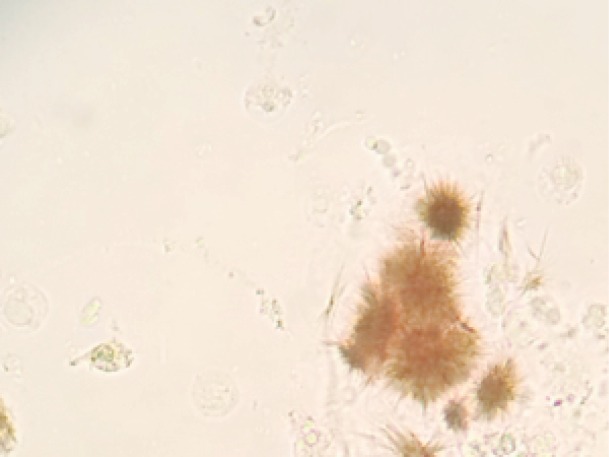
Stellate-shaped ciprofloxacin crystals at high power

**Figure 5:**
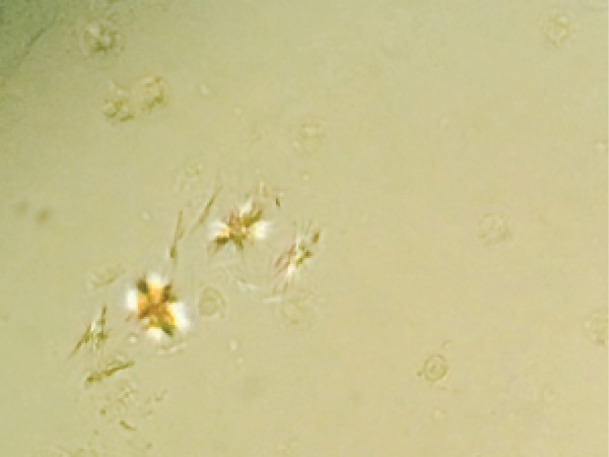
Ciprofloxacin crystals under polarized light

**Figure 6:**
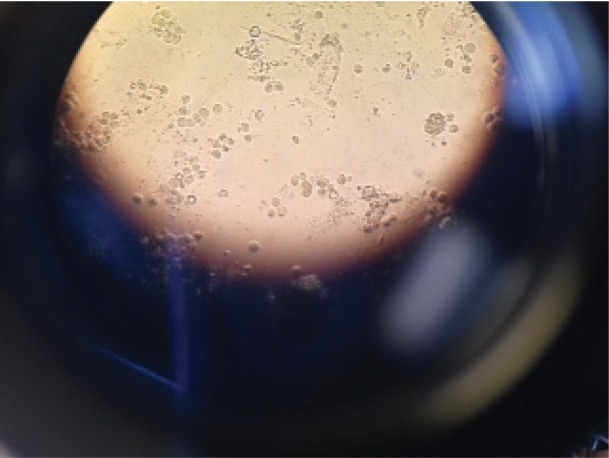
Urine sediment post hydration showing abscence of crystals

The patient is a 72-year-old Caucasian woman with a past medical history significant for metastatic adenocarcinoma of the colon status post chemotherapy with FOLFIRI that was completed three months prior to admission. Previously she had an episode of bacteremia due to colitis and transient acute renal failure (ARF) requiring temporary renal replacement therapy. She presented with nausea, vomiting and abdominal pain. A non-contrast enhanced CT showed thickening of the rectosigmoid colon and possible proctocolitis. Serum creatinine (SCr) on admission was 0.91 mg/dl on day 1, increasing in the next 24-48 hours to 1.2 and 2.4 mg/dl, respectively. Patient received oral (500 mg BID) and later intravenous (400 mg BID) ciprofloxacin on days 1 and 2, stopped on day 3. The antibiotic regimen was changed to metronidazole after documented *Clostridium Difficile* infection in the stool culture. Serum creatinine continued to rise progressively up to a value of 3.54 mg/dl on day 7 (Figure 1). These increases coincided with a constant decrease in urine output below 500 cc for 6-8 days (Figure 2). We were consulted on day 6. Urine sediment showed stellate shaped crystals (Figure 3 and 4) that were birefringent to polarized light (Figure 5) and highly suspicious for ciprofloxacin crystals. Aggressive hydration with 0.9% NaCl was initiated avoiding alkalinization of the urine at all times (average urine pH=5, pH=7 on presentation) despite using sporadic infusions of sodium bicarbonate to treat worsening academia due to renal failure. Creatinine values improved in the following days down to 1.81 mg/dl. Repeat urine sediment examination showed complete resolution of crystals with sporadic granular casts (Figure 6) that probably contributed to the slow recovery in renal function. Patient fared well and was discharge uneventfully to her nursing home.

## 3. Discussion

We report an elderly woman with documented ciprofloxacin crystal-induced renal failure. This represents and addition to the few cases published in the literature in recent years. Ciprofloxacin can cause crystalluria in alkaline urine especially at pH>7.3 both in experimental animals and human subjects (8). Reports have shown crystal deposition in the renal tubular lumen even with a pH < 6.8 (9). Some case reports have also documented crystal-induced AKI with standard doses of ciprofloxacin during one to 8 days of therapy (6-9). Crystals present in different shapes and forms: needle shaped stellate, “sheaves”, “stars”, “fans” and “butterflies” as well as other unusual shapes all with a lamellar structure whose sizes ranged from 30×5 µm to 360×237 µm. Some crystals are colorless while others have a brownish hue. Under polarized light , ciprofloxacin crystals are birefringent (6). Our patient’s urine sediment demonstrated similar type of crystals as described above. Serum creatinine improved considerably with aggressive hydration, after ciprofloxacin was discontinued. Changes in urine output correlated, to certain extent, with renal function parameters (SCr) and estimated glomerular filtration rates. The fact that the final SCr did not return to baseline probably had to do with the presence of residual ATN. The teaching point is the awareness of crystal induced reversible AKI due to exposure to short courses of ciprofloxacin given orally or intravenously.

## 4. Conclusions

The case also stresses the importance of microscopic examination of a urine sediment (using a polarizer), for the early diagnoses of quinolone induced reversible acute kidney injury, avoiding the need of a kidney biopsy. Treatment is conservative and includes hydration with isotonic or hypotonic solutions and prevention of alkalinization of the urine (7).

## Authors’ contributions

All authors contributed to the manuscript equally.

## Conflict of interests

The authors declared no competing interests.

## Funding/Support

None.
